# Undercompression errors as evidence for conceptual primitives

**DOI:** 10.3389/fpsyg.2023.1104930

**Published:** 2023-04-26

**Authors:** Maria Teresa Guasti, Artemis Alexiadou, Uli Sauerland

**Affiliations:** ^1^Department of Psychology, University of Milano-Bicocca, Milan, Italy; ^2^Leibniz-Centre General Linguistics (ZAS), Berlin, Germany; ^3^Institute of German Language and Linguistics, Humboldt-Universität zu Berlin, Berlin, Germany

**Keywords:** commission errors, relative clauses, negation, event structure, dependencies

## Abstract

The Meaning First Approach offers a model of the relation between thought and language that includes a Generator and a Compressor. The Generator build non-linguistic thought structures and the Compressor is responsible for its articulation through three processes: structure-preserving linearization, lexification, and compression via non-articulation of concepts when licensed. One goal of this paper is to show that a range of phenomena in child language can be explained in a unified way within the Meaning First Approach by the assumption that children differ from adults with respect to compression and, specifically, that they may undercompress in production, an idea that sets a research agenda for the study of language acquisition. We focus on dependencies involving pronouns or gaps in relative clauses and wh-questions, multi-argument verbal concepts, and antonymic concepts involving negation or other opposites. We present extant evidence from the literature that children produce undercompression errors (a type of commission errors) that are predicted by the Meaning First Approach. We also summarize data that children’s comprehension ability provides evidence for the Meaning First Approach prediction that decompression should be challenging, when there is no 1-to-1 correspondence.

## 1. Introduction

When children acquire language, nature and nurture interact. A theory of the acquisition process must therefore make assumptions about the child’s human biological endowment, including the child’s sensitivity to aspects of its environment. In this paper, we approach this topic from the point of view of the Meaning First Approach (MFA), as presented in Sauerland and Alexiadou (2020), (in print). Our goal is, on the one hand, to show that certain errors uncovered in the literature on child language can be offered a unified account with the assumptions of the MFA and, on the other, to present a research agenda for using child data in the development of a new perspective on the relation of thought and language. As a first step, in this section we briefly summarize the main features of the MFA, pointing out similarities and divergencies with respect to other approaches. In doing this, we will avoid the technical jargon and we refer the reader to Sauerland and Alexiadou (2020), (in print) for more formal details. Although we build on previous research on model theoretic semantics and generative syntax-morphology, the assumptions and predictions of the MFA differ from that of these other approaches to language, including mainstream generative grammar and the minimalist program. One difference is that, while the other proposals assume that structure building is an operation in language, Merge (Chomsky, 1995; Jackendoff, 2007; Everaert et al., 2015; and others), the MFA assumes that structure generation is independent of language. Specifically, the MFA holds that structure building takes place in a system called the “Language of Thought” in previous literature. In this respect, the MFA capitalizes on the primacy of meaning. A second difference is the assumption that language allows associating meaning directly to sounds and signs, unlike the T-model and systems like Parallel Architecture (Jackendoff, 2007). In line with these assumptions, the MFA postulates a model in which there are two systems: A Generator and a Compressor. The Generator, located in the Thought-system, is the structure-building engine that is responsible for the generation of complex thoughts that we call Conceptual Representations (CR), which are the meanings of sentences. CRs are generated out of universal and innate primitives (core primitives) and primitives derived from experience (non-core primitives) through algebraic operations. Sauerland and Alexiadou (2020) essentially adopt the operation Merge of the minimalist program (Chomsky, 1995) as the algebraic operation and if this is correct, CRs are unordered binary branching tree structures. The generator is independent of language, universal within the species and is at least partly shared with other non-human species. Possible core primitives and compositional relations are causation, agency, conjunction and negation (Carey, 2009). The generated CRs are mental objects that represent the meanings one wants to express and, in this respect, are universal except for the non-core concepts. A central hypothesis of the model is that CRs are rich and complex, and not being designed for communication, may include redundancies that need not be expressed linguistically for successful communication. In order to share CRs, humans, but not other species, use language, which, instead, may be influenced by communicative restrictions and information theoretic cost/benefit considerations, which lead to compress the richness and redundancies of the CRs and thus not articulate what can be highly predictable. It is conjectured that language relates CRs to sounds and signs directly through the Compressor. The Compressor is responsible for choosing one of the possible linguistic form of articulation or externalization. In fact, there may be multiple ways to externalize a complex, rich and redundant conceptual structure. For example, one could say “John cleaned the house, because Paul was going to visit him” or “John cleaned the house and the reason for this was that Paul was going to visit him” or “Paul was going to visit John, who therefore cleaned his house” to externalize a complex representation that causally connects two thoughts. In this respect, the externalization occurring through the Compressor differs from externalization occurring in current approaches such as Minimalism. In these approaches, externalization is fully deterministic, while a choice must be operated in MF. Choosing a form of externalization involves dealing with linearization, the choice of which parts of the CRs realize (i.e., articulate) and the choice of lexical items. One way to formally implement compression is through the exponence relation of Distributed Morphology (see Halle and Marantz, 1993). Compression is guided by various restrictions that interact with each other, such as information-theoretic and communicative considerations, the context of use, the linguistic items, rules available in a given language, and considerations of learnability, so that what is realized must be the least material as necessary to recover the meaning that is intended to be conveyed by a particular CR, to a sufficient extent. As a first approximation, this means that whenever ellipsis is possible up to the recovery of meaning, it must apply (provided that what results is still learnable). Otherwise, a manner implicature or ungrammaticality arises. Sauerland and Alexiadou (2020) give the example of present tense expression in English in (1). In this example, we avoid a complex formalization. In (1a), we have the CR (in capital letters are the concepts structurally organized in brackets, with the structure given by merging concepts), and in (1b), it’ articulation, with tense not being pronounced. But if tense is articulated as in (1d), a manner implicature (Grice, 1989) is observed and the CR corresponding to (1d) must include a focus concept, as in (1c).

**Table:** 

(1)	a.	[EFFECTOR [PLURAL PRESENT [LIKE LINGUISTICS]]].
	b.	We like linguistics.
	c.	[EFFECTOR [PLURAL [PRESENT FOCUS]] LIKE LINGUISTICS].
	d.	We do like linguistics.

Implicit in the idea of compression of CRs is the debated assumption that there are few 1-to-1 correspondences between CRs and linguistic expressions and this represents a third deviation from previous literature (such as Montague, 1970; Langacker, 1987; Fodor, 2008). A 1-to-1 mapping would be needed if every element of a CR needs to be communicated or in other words, if CRs and their articulations into language would be subject to the same constraints, but this is unlikely. First, while language is used to communicate in an efficient way and thus is subject to principles of optimization of information theory (Shannon, 1948; Hale, 2016), CRs are not suited or organized for communication (Chomsky, 2015). Second, language unfolds in time and needs to be linearized, while CRs do likely not include a temporal dimension and certainly do not need to be linearized. Third, primitive concepts are universal, while the linguistic means of lexicalization (linguistic expressions) vary and accordingly the specific way of compressing differ across languages. These observations together with the assumption that CRs are richer than what is expressed into language conjure up the conclusion that each individual language only ever reflects fragments of any CR. This basic tenet of the MFA that CRs are richer (and thus redundant) than their corresponding linguistic expressions defines a research agenda. From the MFA perspective, we need to undo compression, which is what is available or we have access to, to determine CR’s primitive units and their combinations into complex concepts. But how can we do this? To answer this question, we adopt an approach that has been very fruitful in the history of cognitive science: investigating situations in which a given system is not fully functioning, in our case a system that has not yet acquired all the lexical means of lexicalization and the specific constraints of compression operative in a given language. One may learn how a non-impaired system works by looking at situations where there are some local breakdowns, such as, e.g., in aphasia. By looking at symptoms in these situations (e.g., one patient uses inappropriate nouns and another one verbs) through the lens of some theoretical background, one may learn how the non-impaired system is structured. In the following section, we argue that child languages are a particularly illuminating case of an incomplete system for the MFA, and allow us to overcome compression, at least in some cases. Then, we examine the evidence for the predictions of the MFA that children’s utterances are richer than those found in the target language to express a given conceptual structure. We also present existing evidence in favor of the prediction of the MFA that comprehension of language should be more accessible when sentences are closer to the conceptual representation, i.e., more transparent. In so doing, the MFA postulates that children’s languages are the best mirror of the human mind. Finally, we discuss alternative approaches to the empirical data presented in the previous section.

## 2. Meaning first meets language acquisition

The MFA assumes that primitive concepts are innate, universally needed independently of language and partially shared across non-human animals. The compositional rules are also innate and necessary to combine primitive concepts into complex, structured thoughts. This ensures the generation of structured conceptual representation, whose existence is needed independently of language. In other words, the MFA assumes that structured meaningful representations can be available to a child (as well as non-human animals) before learning a language. A recent review article by Quilty-Dunn et al. (2022) discusses several pieces of evidence from both infants and non-human animals’ studies, among other data, in favor of a language of thought as one of the representational formats of cognition and thus in line with our view that structured meaningful representation can be available independently of language. In fact, research has accumulated showing that infants, as young as 4-month-old can use abstract content and reason about it under certain conditions (when their attention is attracted to the relevant dimension through priming, e.g., Lin et al., 2021 or when some categories are relevant for them or salient, e.g., Bonatti et al., 2002; Surian and Caldi, 2010), suggesting that failures noticed in the literatures are due to testing conditions, rather than to lack of competence (Stavans et al., 2019). Twelve month-old infants may perform logical inferences using logical operators, as in the disjunctive syllogistic reasoning (Cesana-Arlotti et al., 2018, but see Leahy and Carey, 2020). Similar abilities have been uncovered in animals. Bumblebees and honeybees can have abstract representations of some features (dimension or numerosity) and use them in inferential activities (e.g., Gallistel, 2011; Solvi et al., 2020; Weise et al., 2022). Chicks display the capacity to abstract even shortly after birth [Wood and Wood (2020); see also Bermúdez (2003) for the presence of some abstract concepts in animals]. Like infants, an African gray parrot can perform logical inferences (Pepperberg et al., 2019). All in all, these studies demonstrate that preverbal infants as well as non-human animals can have abstract concepts, structured representations and can use them in reasoning, as assumed in the MFA. A further assumption of the MFA is that a compression function compresses the conceptual structures generated into language.

These assumptions lead us to propose that what the children have to learn from experience is the inventory of non-core primitive concepts, the mapping between primitive and complex concepts and linguistic expressions (morphemes, prosodic features), and the compression function, which comprises linearization and articulation rules for a given CR. They also have to learn the pragmatic contexts that make language utterances felicitous and the coordination of linguistic means and contexts. The process of learning how to compress in a given language may take time. Specific cases of compression may depend on advanced knowledge of the language use and pragmatic understanding, including avoidance of redundant items, and all this may not available to the child when she starts to produce multiword sequences. Does the idea that thought structures are compressed into language have to be learned by a child? We believe that if it does, it is something that can be learned in the first year of life through communicative interactions. First, in the first months of life, children communicate with means other than language, e.g., gestures, vocalizations. When language starts to be used, they already know that they can also share some aspects of their thoughts through other means and that language may be combined with these other means, and therefore, not everything needs to be expressed into language. In addition, language and thought are differently constrained, as mentioned earlier. At the same time, children do not know what needs to be expressed linguistically, what can be provided by other means, what is redundant and what can be recovered without articulation, i.e., they do not know how compression works in their language. We argue that these conjectures predict certain properties of child language that have been noted in prior literature but not derived systematically from a unique framework. In particular, children are expected to at least occasionally use language creatively, namely in cases where they have a CR in mind but have not yet fully acquired the adult way of expressing it in a particular language. They may create alternative and more verbose (in comparison to the adult target) ways to express it. We will refer to such creative uses of language by children as *Undercompression Errors* (these productions are one type of non-target productions belonging to the family of commission errors as opposed to omission errors, failure to produce some piece of utterance).^1^ Undercompression Errors are ‘errors’ whereby the child expresses a complex concept *C* with more exponents than an adult would do or would use redundancies staying for concepts present in the CR, but not necessary to recover the intended meaning.^2^ Undercompression Errors originate from bias and learning strategies that guide children in figuring out how the compression function operates. One of these learning strategies that has been proposed in the literature is that children are biased toward a transparency principle (Slobin and Bever, 1982; Jackendoff, 1990; Weist et al., 1997; Van Hout, 1998). In our system, this principle follows naturally from our approach in that children have to map CRs to linguistic expressions; therefore, it is natural for them to be biased toward a 1-to-1 correspondence between CRs and sentences of a given language because this bias avoids ambiguity. Here, we adopt van Hout’s formulation:

(2) Transparency Principle

If acquisition involves finding the mappings between particular concepts and their linguistic encodings, […], then learning should be easier for overt and unambiguous mappings (one-to-one) than for covert and/or conflated ones (many-to-one) (Van Hout, 1998, p. 399).

According to this principle, children comprehend earlier the linguistic realizations of a concept that is unique to it and not shared among concepts (many-to-one).^3^ We will offer examples to illustrate this bias while discussing the evidence for our acquisition approach. But we also are interested in a further prediction of (2) in the context of the MFA to child language production. We anticipate that this principle leads to specific errors of undercompression, i.e., utterances in which children produce more than adults would do or recruit morphemes to express a certain meaning. For example, Ilić et al. (2022) have proposed that English-speaking children produce a redundant (verb) DO in affirmative sentences to express Agency. They cite other child languages where this has been observed. Their claim is motivated by the fact that these uses of redundant DO were only found with agentive verbs, i.e., verbs that already encode in a compressed way the information of agency. Nevertheless, children articulated an item to specifically encode in a transparent way the agency concept. English-speaking adults do not use the verb DO to articulate agency, as the concept agency can be recovered from the meaning of the verb; however, they use the auxiliary DO to express Tense or focus (see ex. 1 and 2 above). We hypothesize that the expression of agency through DO, along with other errors that we are going to discuss, is an undercompression error and formulate this hypothesis in (CLUH):

(3) Child Language Undercompression Hypothesis

If concept C occurs in a conceptual representation and C can be realized by a non-null morph M in some environments by adults, children will realize C as M in all environments at non-zero frequency.

We recognize that in this form, the statement may be too strong, and there may be restrictions on undercompression, even for children. However, there is considerable evidence that undercompression Errors do exist. If constraints on child undercompression are found in future research, they need to be added to our hypothesis.

Based on the CLUH, the challenge of overcoming compression can be addressed by investigating the languages of children. Adult languages compress thought structures and eliminate redundancies for communicative purposes and economy considerations. Moreover, compression may take different forms depending on the type of morphological means available in the specific language. Finally, within the same language, some areas may be more transparent than others. Therefore, it may not always be easy to recover CRs, although, we do not deny that cross-linguistic investigation may offer some insights. At the same time, we think that cross-linguistic investigation of child language may offer even more insights. In fact, we predict that children availing themselves of a not fully functional system are less able to compress and, being biased by the 1-to-1 mapping are more likely to produce sentences that are closer to CRs and are revealing of their structures. In other words, we bet that there are areas where children systematically use non-adult/non-target sentences, in which they encode through linguistic expressions more concepts than needed for communication and, we conjecture that these non-adult sentences reveal pieces of CRs. Thus, we anticipate that we will recover evidence for how CRs are structured by looking at the languages of children around the world (or other learners, adults, while they are learning), i.e., at systems of knowledge not yet stable or not yet fully functioning from the point of view of the adult/target system.

### 2.1. Specific areas of focus

This paper presents preliminary evidence in favor of the child language undercompression hypothesis (CLUH) within the MFA. Testing the prediction that unpronounced pieces of a CR can be pronounced in the child language requires a commitment to the primitives and relations of CRs. At this point, though, we only have a partial understanding of CRs, so our research program involves an interplay starting with predictions based on our partial understanding of CRs and then using initial results from child language to foster our understanding of CRs. The areas of language we initially focus on are three domains where independent work on the syntax-semantics interface has provided evidence that the meaning of certain words or phrases can be decomposed into separate parts that are not transparent from the morphology. In particular, we focus on three domains: variable binding dependencies, verbal concepts relating to multiple arguments, and antonymic concepts such as existence vs. non-existence. In the following sections, we briefly outline what is known about the CRs of dependencies, multi-argument concepts, and antonymic concepts.

#### 2.1.1. Dependencies

For Dependencies, let us first specify the term since our view about them is explicated by appeal to formal logic. Frege (1879) invented the notion of a bound variable that represents one way to understand dependencies, though alternatives exist (e.g., combinatorial logic of Schönfinkel, 1924). In English, there are primarily two ways a bound element may be articulated: as a gap/trace (i.e., not pronounced at all) or as a pronoun. The presence of a dependent element in (4a) is indicated by the stilted English paraphrase (4b) involving *the dog* in both the trace and the pronoun position and by the representation in (4c) in the style of Kratzer and Heim (1998) that invokes actual Fregean variables.

**Table:** 

(4)	a.	Every dog Anna petted ___ wagged its tail.
	b.	Every dog such that Anna petted the dog wagged the dog’s tail.
	c.	Every dog [Op_1_ Anna petted t_1_] [Op_1_ t_1_ wagged it_1_’s tail].

It has been argued that both traces and pronouns must be complex concepts and share some lexical material with the binder [i.e., the representation in (4c) is inaccurate]. For traces, this is known as the copy theory of traces (Chomsky, 1995; Sauerland, 1998, 2004; Fox, 2000). For bound variable pronouns, Sauerland (2000a,2008) argues in favor of the same point. Also relevant is the work on so-called donkey pronouns that cannot be directly captured as bound by an overt phrase in the logical sense but have been analyzed via a similar indirect relationship by Evans (1980), Heim (1990), Elbourne (2001), and Sauerland (2007). We take the evidence on traces and pronouns to indicate that overall bound variables must share some primitive concepts with their binder. By this we mean that the concepts lexicalized in the binder and the bindee must totally or partially overlap. As a result, for example, if the binder lexicalizes the concepts “animate” and “dog,” the same may be lexicalized in the bindee. Furthermore, the discussion of traces has led theoreticians to distinguish between so-called A and A’ traces, and Takahashi and Hulsey (2009) have argued that A’ traces have to share more material with their binder than A traces need to, but propose that A-traces also at least share the conceptual information related to nominal category. The A/A’ distinction correlates with the structural distance between a trace and its binder. Thus, the number of primitive concepts shared between the binder and bound element correlates with the structural proximity of the two: the greater the structural distance, the more concepts must be shared. As a result, the binder can identify the relevant bindee, thus incorporating for free some form of locality between dependencies (see Sauerland et al., submitted). Therefore, we arrive at the following generalization as the basis for our investigation of acquisition. In (5), we leave the full domain of application of the generalization open since not all relevant cases have been scrutinized.

(5) Bound element generalization

Within a class of cases that includes at least A’ traces, any concept that can be bound in the sense of variable binding must share one or more primitive concepts with the concept binding it.

#### 2.1.2. Multi-argument verbal concepts

For the meaning of verbs (and possibly of other categories), it has been argued that it can be decomposed into a number of more primitive units, which are hierarchically organized. Specifically, it has been argued that multi-argument verbs include unpronounced light verbs and verbal stem (cf. Rappaport-Hovav and Levin, 1998, 2010; Borer, 2005; Levin and Rappaport-Hovav, 2005; Alexiadou et al., 2015; Ramchand, 2018; among others; see Senghas et al., 1997 for a type of decomposition in the genesis of Nicaraguan Sign Language). Thus, in the case of (6), we have a core dynamic event, CAUSE, that combines with a result component, BROKEN, and a layer above the dynamic event associated with the external argument. By contrast, the internal argument is associated with the result component. In this example, the concept CAUSE is compressed or not pronounced, while the result is exponed by the verb stem. Verbs associated with only one argument do not show such complexity, and (7) simply involves a dynamic event and a layer above the dynamic event associated with the external argument.

**Table:** 

(6)	John broke the window.
	[AGENT [[_dynamic event_ CAUSE] [_Result_ THEME BROKEN]]].

**Table:** 

(7)	John ran.
	[AGENT [_dynamic event_ RUN]].

Rappaport-Hovav and Levin (2001) put forward the one argument per subevent principle, according to which there must be one argument in the syntax to identify each sub-event in the event structure template. We can reformulate this principle in the following generalization:

(8) Multi-argument generalization

Within a class of cases that includes at least causative verbs, any concept that takes more than one argument of the type of individuals must be complex and include light verbs along with a root stem.

#### 2.1.3. Antonymic concepts

Antonymic expressions are found in several different grammatical categories, for example, quantificational determiners *a-none*, verbs *continue-stop*, adjectives *tall-short*, prepositions *inside-outside*, adverbs *sometimes-never*, and nouns *child-adult* (Déprez and Espinal, 2020). For all antonym pairs, there is an intuition that the negation of one member is logically equivalent to the other, e.g., *not continue* is equivalent to *stop*, and also, *not stop* is equivalent to *continue*. But antonym pairs in many cases display also subtle asymmetries. For negative quantificational determiners, Bech (1955) showed that German allows interpretations where a negative part and a positive existential seem to contribute to sentence meaning in different positions (see Penka, 2011). Sauerland (2000b) argued that across languages, the negative existential seems never to be a primitive concept but would need to be composed of negation and an existential. For adjectives, Bierwisch (1967) has have pointed out that there frequently are subtle markedness asymmetries between the two members of an antonym pair. For example, the question *How tall are they? is* more neutral than the question *How short are they?* indicating that *short* is the marked member of the pair. Another way to look at this asymmetry with dimensional adjectives is by noting that while *tall* has no upper bound in principle, *short* must have a minimum size. So, the marked member must have a minimal lower bound, while the unmarked has no upper bound. Looking cross-linguistically, there are cases where the marked member can only be expressed by means of negation of the unmarked member, e.g., the pair *kawo*–*kawo-hra* (“long”—“short,” lit. “long-NEG”) in Hixkaryana (Derbyshire, 1985). These data and other pieces of evidence have led several theoreticians to conclude that one member of antonymic adjective pairs has to be decomposed across all languages into a negation marker and a positive adjective (Heim, 2006; Büring, 2007; Bobaljik, 2012; Rett, 2014; Moracchini, 2019). For prepositions, the English pair *with*—*without* is similar to Hixkaryana if we assume that *out* is a morphological exponent of negation in this domain. Thus, we assume that antonymic pairs of concepts fall under the following generalization:

(9) Antonym generalization

Within a class of cases that includes at least negative quantifiers, dimensional adjectives and *without*, the marked member of an antonym pair is never a primitive concept, but is composed of negation and the unmarked member of an antonym pair.

## 3. Predictions for child language: undercompression errors

A common thread connects the three domains described above: the conceptual representation is richer than the adults’ linguistic realization. Accordingly, the CLUH predicts that children may sometimes produce more concepts from the CR when they express their thoughts, i.e., they may creatively add linguistic material that is not present in the adult utterance (see also Belletti, 2022) or produce undercompression errors. This may happen because children do not yet know the rules of compression in their language, are biased by the 1-to-1 mapping or because of third-factor reasons. In addition, we anticipate that the acquisition of language may proceed more smoothly in cases where there is a 1-to-1 correspondence between the CR and its linguistic expression (Van Hout, 1998 as discussed above, also Culbertson and Adger, 2014 on adults learning an artificial language) and third, children may interpret sentences differently than adults because they apply the 1-to-1 mapping. In other words, the often observed 1-to-1 bias in child language stems from the structure of the CR and the attempt to make the mapping as transparently as possible, because this is meant to facilitate acquisition and the recovery of meaning.

Thus, we conjecture that undercompression errors are revealing pieces of the CRs that are compressed in the adult language. Since some languages may not compress a given CR or never express some primitives, while others do, it is apparent that if we want to recover pieces of the CR from child language, a single child language is not enough. It may reveal one piece, but other pieces will be missed, because the target language does not compress in the first place. To recover as much as possible of the CR, we need to adopt a cross-linguistic approach and look at as many child languages as possible. It is only this cross-linguistic comparison that will enable us to put together the pieces of the puzzle, as it will appear clear from the cases that we will illustrate. If adult language reflects a fixed, partial view of the CR, child language can be compared to a wobbly mirror that can lead to new insights. Specifically, on the basis of the generalization (7), (8), and (9) we formulate the following three predictions:

**Prediction 1**: In cases where a bound element entering a variable binding dependency shares one or more primitive concepts with the element binding it, children are expected to produce “filled gaps.”
  **Prediction 2**: If a verbal concept has as part a null light verb that has a pronounced equivalent, children are expected to pronounce the light verb.
  **Prediction 3**: If one member of an antonym pair is decomposed into negation and its antonymic counterpart, children are predicted to produce a bimorphemic antonym structure.

In the rest of the article, we are going to develop our argumentation with examples reported in the literature from child language focusing on the three domains—dependencies, verbal arguments and antonymic concepts–and showing how children’s production can corroborate our understanding of the CR, and can therefore serve to guide future research.

### 3.1. Dependencies

In this section, we are going to discuss two domains that test the predictions of the CLUH in relation to our generalization concerning dependencies, that is, the fact that the binder and the bindee in a variable binding relation must share one or more concepts. The two domains are relative clauses and long-distance questions, for which substantial evidence favors undercompression (further predictions concern E-type pronouns and comparatives that will be addressed in future work).

#### 3.1.1. Relative clauses

The generalization concerning dependency in (5) holds that the binder and the bindee must share one or more primitive concepts. In the light of this, the CLUH predicts that children may sometimes expone not only the binder, but also the bindee or some concepts forming it. Underlying this prediction is the assumption that relative clauses results from the assembly of several complex concepts. Let’s illustrate this assumption in an informal way by considering (10). One piece is the CR underlying “the uncle” and another piece is the CR underlying “the boy hugged the uncle.” When these two pieces are put together, as in a relative clause, a dependency is created with the binder and the bindee being identical, as in the CR in (10), which contains two heads sharing concepts. We further assume that the relative head, N-root, as all nouns, is accompanied by a classifier (CL), which contain functional concepts characterizing the category noun; concepts relevant for agreement may occur as CLs without articulation, while adjective modifiers would be articulated (see below for evidence supporting this assumption). Similarly, the bindee includes the N-root and a classifier. Kayne (2007) and Cinque (2020) develop similar conceptions, though they generally seem to adopt only a single classifier. At this point, we leave underspecified whether (10) corresponds to a raising or matching structure in the sense of Sauerland (1998, 2000c) and Hulsey and Sauerland (2006) since both are predicted to have a double-headed structure. Thus, in this representation the binder, the relative head, and the bindee, the double in the internal position, share concepts, in agreement with our generalization in (5). When this representation is articulated into language, typically only one head is articulated (the internal or external). In some cases, both heads are articulated (double headed RCs), and in this case, one can be the noun and the other a pronoun or again a noun, that is, the double head can be identical to the first or can articulate less concepts.

**Table:** 

(10)	The uncle which	the boy hugged [x — uncle].
CR:	The [[CLs N-root]	Operator-x [x CLs N-root]].

The above CR is supported by comparative research on adult languages. Cinque’s 2013; 2015; 2020 typological overview describes head-external relative clauses, head-internal ones, and actual double-headed relatives (see also Dryer, 2005). Double-headed relative clauses, in turn, display variation in line with our assumptions discussed earlier. In some examples, the relative head in both positions features the same noun (though the relative head contains a demonstrative and a classifier, while the double includes just a bare noun), as in (11) from Ronghong Qian (Tibeto Burman family) (cited in Cinque, 2020 from Huang, 2008) and (12), from Wenzhounese, a language spoken in South-East China (Hu et al., 2018):

**Table:** 

(11)	[[zə**p**	iҩtҩimɑqɑ	ʐɑwɑ tshu-tshu]RC (-tҩ)	**z**ə**p** tha-kua]_NP_.
	[[**place**	usually	rock drop-REDUP(-GEN)]	**place** that-CL].

“The place where rockslides often occur.”

**Table:** 

(12)	ŋɑ^52^ bo^21^	ɦo^31^	mᴈ^33^	k ŋ^33^ kəʔ^0^ hai^33^	kai^42^	mᴈ^42^ ŋ^33^.
	grandma	draw child		REL DEM	CL	child.

“the child that the grandma draws.”

In yet other languages, however, the external head is a general term, while the internal head is a more specific expression. In the example below from Kumbai (*Trans*-New Guinea; Papua, Indonesia, cited in Cinque, 2020), the external head is *ro* “thing” and the internal one is *gana* “bush.knife.”

**Table:** 

(13)	[Gana	gu	fali-kha	]	ro
	[Bush.knife	2SG carry-go.2SG.NONFUT]			thing
	na-gana-y-a.
	my-buah.knife-TR-PRED.

“The bush.knife that you took away, is my bush knife.”

The reverse is found in the Japanese example in (14) (cited in Cinque, 2020, from Kuno, 1973), where the internal head is the more general term and the external one the more specific one.

**Table:** 

(14)	[[watakusi	ga	**sono ito** no	namae o wasurete-shimatta]
	[[I	NOM	**that person**’s	name ACC have forgotten]
	**okyaku-san**].
	**guest**].

“A guest whose name I have forgotten.”

The presence of general and more specific terms in RCs is an indication that nouns are always accompanied by classifying material, CL, and what happens in the last two types of languages is that either the classifying material or the noun is articulated in head or gap positions. Indeed, Kayne (2007) and Cinque (2020) argue that nouns are always associated with functional nouns (N) that classify them, e.g., [_*FP*_ [_*DP*_ guest] person], [_*FP*_ [_*DP*_ chair] furniture]. Typically, these functional nouns are not articulated or are dropped. However, Cinque notices that there are cases in which they are retained as in *New York city*, which alternates with *New York*.

Relevant to the prediction emanating from the CLUH are primarily languages in which relative clauses feature the presence of a gap and an external head in the adult language. And this prediction is that children may produce lexical material in the gap position that shares feature with the relative clause head. In other words, given the CR in (10), they may produce relative clauses in which the head and the double can be identical or either one be a general or a more specific term. This prediction is borne out by several elicited production studies conducted in a variety of early languages with children from age 3;0 up to age 10;0. First, children produce relative clauses featuring the presence of a resumptive pronoun in the gap position (or close to it in languages with clitic pronouns). Second, children produce relative clauses, where the gap is filled with the same material as the relative head, i.e., with resumptive DPs. Third, children may use a more specific term in the external position than in the gap position or the reverse. These productions have been found in Catalan (Gavarró et al., 2012) European Portuguese (Costa et al., 2014), English (Pérez-Leroux, 1995 citing Finer, 1992), French (Ferreiro et al., 1976; Labelle, 1990; Guasti et al., 1996), German (Yatsushiro and Sauerland, 2018), Hebrew (Armon-Lotem et al., 2005), Jakarta Indonesian (Tjung, 2006), Italian (Guasti and Cardinaletti, 2003), Mandarin (Hsu et al., 2009; Hu et al., 2016), Palestinian Arabic (Armon-Lotem et al., 2005), Serbo-Croatian (Goodluck and Stojanovic, 1996), Spanish (Ferreiro et al., 1976; Pérez-Leroux, 1995), Turkish (Özge et al., 2010). Relevant examples are below:

Relative clauses with resumptive pronouns.

**Table:** 

(15)	a. the one that he lifted it (L, 4;5) (Pérez-Leroux, 1995).	
	b. Sur la balle qu’i(l) l’attrape (LE 3;08) (Labelle, 1990).
	“On the ball that he catches it.”		
	c. mama qin wo de xiaopengyou (Hu et al., 2016).	
	mother kiss me DE child.	
	“the child that the mother kisses me.”	

Relative clauses with resumptive-DPs with (almost) the same DP, general and specific terms.

**Table:** 

(16)	a. el gato empuja al perro que el conejo lava al perro. (5;0) (Ferreiro et al., 1976).
	the cat pushes the dog that the rabbit washes the dog.
	b. das Mädchen sein, das der Opa **das Mädchen** umarmt (Yatsushiro and Sauerland, 2018).
	“be the girl who the granddad hugs **the girl**.”
	c. mama qin xiaopengyou de xiaopengyou (Hu et al., 2016).
	mother kiss child DE child.
	“the child that the mother kisses (the child).”
	d. **Celle** que la maman a (= elle) rêve à **une maison** (V 3;11) (Labelle, 1990, 100).
	The one that the mother she is dreaming of a house.
	“The one (= the house) that the mother is dreaming of a house.”
	e. The **one** that the mailman is holding **the baby** (4;11) (Finer, 1992).

Although both resumptive pronouns and resumptive DPs are present in several early languages, resumptive pronouns seem to be more frequent in early languages like French. Labelle (1990) reports that French-speaking children produce 33% resumptive pronouns and 16% resumptive DPs. However, the resumptive pronouns advantage holds for the direct object, locative and genitive RCs; a resumptive DPs advantage is observed for indirect object RCs. Resumptive DPs are more frequent than resumptive pronouns in early Mandarin, but only subject and object RCs were elicited in this case (Hu et al., 2016). Thus, a systematic investigation of these resumptive elements is certainly more than needed (see Guasti and Shlonsky, 1995 for a discussion of the two types of resumption in children’s RCs). We can notice that resumptive relatives with pronouns are common in a variety of languages (Hebrew, Palestinian Arabic, Welsh) and in many colloquial varieties of Romance (French, Italian, Spanish). Thus, in several languages, children hear resumptive relative clauses, along with relative clauses with gaps (Guasti and Cardinaletti, 2003 and reference cited there).

Although the presence of relatives with resumptive DPs has been noticed in several studies, they have been the focus of interest in three studies by Hu et al. (2018) on Wenzhounese, Hu et al. (2016) on Mandarin and Yatsushiro and Sauerland (2018) on German. As noted above, Wenzhounese allows resumptive DPs (along with gaps) also in the adult language, while German and Mandarin do not. Thus, we will report on the last two languages, both using the same elicitation method (Novogrodsky and Friedmann, 2006). Resumptive DPs are massively used in object RCs and sometimes also in subject RCs (50% of the non-target responses) by 6 groups of Mandarin speaking children ranging in age from 3;0 to 8;0 (Hu et al., 2016) (along with some resumptive pronouns, 21% of the non-target responses). Interestingly, monolingual Mandarin-speaking adults also employ resumptive DPs, but only in their object RCs (56%) during the experiment (Hu et al., 2016), although Mandarin does not feature any kind of doubling RCs. In addition, Mandarin-speaking adults accept subject and object RCs with resumptive DPs, although to a lesser extent than RCs with a gap (Hu et al., 2016). Similarly, Yatsushiro and Sauerland’s (2018) found that German-speaking children (age range 4;6–8;7; mean age 6;6) have no trouble in producing target subject RCs, but produce few target object RCs (18%) and most of the time (41%) produce resumptive DPs along with resumptive determiners/pronouns in this case, as in (17).

**Table:** 

(17)	Ich	möchte bei	den Kindern sein, die	die Oma
	I	want	among the children be,	
	die Kinder malt.
	who-PL the grandma the children draws.

“I want to be among the children that Oma malt the children.”

German and Mandarin feature two different types of RCs: head initial and head final. Yet, children in both languages use DP-resumption, indicating that this is clearly an option for them, although at this point we cannot tell whether it is more frequent in one or the other language and we do not have adult data from German.

In sum, child language provides evidence that in uttering relative clauses children may sometimes realize more of the CR than in their target model. In fact, not only children but also adults can realize it on occasions (e.g., when relative clauses are more complex, as object relative clauses are). Although at this point we lack evidence, we may conjecture that input may modulate the amount and type of possible realizations; nouns or pronouns may be more or less used by children depending on the language.

#### 3.1.2. Long distance Wh-questions

Another structure involving a dependency and for which we predict that children may undercompress are wh-questions. Recall that according to our generalization, the binder and the bindee in a dependency must share some concepts. For a monoclausal matrix question this is illustrated by (18).

(18) Which cookie did the donkey eat which cookie?

We therefore might expect that children should articulate “which cookie” in the position following “eat” at some stage of development. But it is also possible that children may have sufficient cognitive resources to avoid this type of undercompression error from the start of their use of questions. And in fact, to our knowledge undercompression errors with monoclausal matrix questions haven’t been reported. But, for more complex questions involving extraction from an embedded clause, undercompression errors have been reported. Three-to-six-year-old children speaking Dutch (van Kampen, 1997; Jakubowicz and Strik, 2008), English (Thornton, 1990), French (Oiry and Dermidache, 2006), Spanish and Basque (Mangado, 2006) have been found to produce non-target structures in their language of exposure like in (19), featuring *wh-copying* of the wh-word (McDaniel, 1986; examples from Thornton, 1990).

**Table:** 

(19)	Who do you think who Grover wants to hug? (TI 4;9).
	What do you think what Cookie Monster eats? (KM 5;5).

In (19), the wh-phrase content isn’t articulated in the argument position of the verb, but in an intermediate position. As far as we know, articulation in the argument position has not been found in child language for languages that are not adult wh-*in situ* languages in neither monoclausal nor long-distance questions. Our proposal for the analysis of (19) as undercompression errors adopts the assumption that cognitive representations minimize the length of dependencies in a non-additive fashion (Fox, 2000; Sauerland, 2018). This implies that a CR with two short dependencies is preferred over a CR with one dependency that has double the length of the short dependencies. This assumption leads us to postulate the following preliminary CR if an intermediate position for a wh-phrase is available [QUESTION would mark the clause as a question, while we understand *who* (analyzed as WH PERSON) here as an indefinite element]. So (20) could be paraphrased as “*Some person’s identity is relevant and you think that Grover wants to hug that person”*:

**Table:** 

(20)	[[[WH PERSON] QUESTION]
	[you think [[WH PERSON] Grover wants [hug [WH PERSON]]]]].

Adult languages differ in what of this CR is articulated and how. In several languages only one set of concepts is articulated. Thus, a question extracting from an embedded clause looks like in (21), where the wh-word/phrase fronted in sentence-initial position conflates the concepts WH-N and QUESTION and the intermediate WH-N concept is not articulated.

(21) Which boy/Who do you think that Grover wants to hug?

But children may sometimes pronounce the medial wh-position, resulting in productions like those in (19) above, where they spell out the two short dependencies.

There are also adult languages, where two sets of concepts can be articulated. In fact, there are two varieties of such strategies: Wh-copying, such as in Afrikans, dialects of German, Dutch, Frisian, and Romani (McDaniel, 1986; Felser, 2004) and scope marking (or partial movement strategy), such as in dialects of German, Malay (Cole and Hermon, 2000), and Hindi (Dayal, 1996). So, even adults in some languages may undercompress. But the fact that the undercompression is spontaneously produced by children acquiring an adult language that doesn’t have the structure supports our proposal.

We also predict that children may produce some material in the place of the gap. This type of production has not been reported so far in the literature, but preliminary evidence from Italian seems to suggest that children can sometimes express an indefinite (someone) in the gap position (Dal Farra et al., 2022a).

Another prediction is that not only children will attempt to produce in a more transparent way the CR, but they will interpret sentences through the 1-to-1 mapping principle and, in this respect, they may assign to a sentence a different interpretation than an adult would. de Villiers et al. (2008) found that children may interpret questions like (22) as scope marking questions and answer the medial indirect question (“how she ripped her dress”), rather than the matrix question. Hence, they may answer with “with the wire on the fence.”

(22) When did you say how she ripped her dress?

These types of errors of interpretation indicate that children take the embedded wh-word as the element introducing the real question and the fronted one to be a scope marker. In our framework, these answers are the mirror image of what children do in production and their source is the attempt to transparently map the CR. From 4 to 5 years, we observe a decline of these “medial” answers, which disappear by age 7 (see Guasti, 2017 for review). Interestingly, these ages match the age in which wh-copying and medial wh-questions are produced, suggesting that their source is the same, the use of the transparency principle.

### 3.2. Multi-argument verbal concepts: causative verbs

We now turn to the second domain we identified, multi-argument verbal concepts and illustrate our generalization that any verbal concept that takes more than one argument of the type of individual must be complex and thus include light verbs along with the verb stem; in other words, such verbal concepts include multiple subevents. We will illustrate this through lexical causative verbs.

According to our generalization, the underlying CR of a sentence including a lexical causative verb contains a CAUSE and RESULT concept as in (6) repeated in (23). Each of these concepts introduces one argument, the Effector (or Agent or causer) introduced by the concept staying for a light causative verb and the patient introduced by the concept staying for the resulting state.

(23) [EFFECTOR/AGENT [[_dynamic event_ CAUSE] [_Result_ THEME BROKEN]]].

Generally, when the CR in (23) is articulated the two concepts are compressed into a single verb. However, Martin et al. (2022) observe that, although redundant causatives are not found in Standard French, they are found in non-standard varieties of French. Similarly, redundant causatives are attested in colloquial Hebrew (Doron, 2003), and in several standard languages, such as Turkish (Göksel, 1993), Hungarian (Hetzron, 1976), and Kashmiri (Manetta, 2014). As in previous cases, the CR in (23) leads us to expect that children, who are biased toward a transparent mapping occasionally articulate both concepts. Anedoctic evidence for this prediction was reported in Aksu-Koç and Slobin (1985), based on the speech of Turkish-speaking children. Recently, this phenomenon has been systematically investigated by Martin et al. (2022). These authors found that French-speaking children produce lexically causative verbs accompanied by an additional redundant causative verb, *faire* (make) as in (24). We call these instances double causatives. From the context, it was clear that sentence (24a) meant “we are going to cut it” and not “we are going to make it cut.” In (24b), we have a second example, which illustrates that the child has acquired the target lexical causative verb, but at the same time adds an extra causative verb *faire* to convey the same meaning (examples from Childes, MacWhinney, 2000). Martin et al. (2022) also showed that children are not mistakenly using transitive causative verbs like “cacher” (hide) intransitively and thus using *faire* to turn them into transitive verbs.

**Table:** 

(24)	a. Va le **faire couper.**
	Go it make cut.
	“Going to cut it” (Anais, 2;9, Lyon).
	b. On va le chacher. On va le faire cacher (Madeleine 2;2).
	We will hide him. We will make it hide = we will hide.

In the relevant period during which children produce these double causatives, they also produce target-like analytical causatives introduced by the verb *faire* with any type of verb (causative and non-causative transitive, unergative, unaccusative verbs), as illustrated in (25).

**Table:** 

(25)	Je vais le faire monter (David 45, 3;09).
	I will it make climb.
	“I will make it climb.”

Interestingly, instead, double causatives were mostly produced with causative transitive verbs. These productions are found from the age of 2;0 and persist till age 4;6. They disappear around age 5;0.

Beyond the production of pieces of the CR not articulated in adult language, we predict that children’s interpretation of causatives may also be guided by the 1-to-1 mapping and this may be responsible for some non-adult interpretation. In fact, this is indeed the case, as shown by the comprehension of Japanese morphological causatives. Yamakoshi et al. (2018) tested the comprehension of morphological causative sentences, as in (26) including the morpheme –*sase*. (26) means that the monkey had someone open the box. Instead, between 4 and 6 years of age children interpreted this sentence as meaning that the monkey itself opened the box 60% of the time; that is, they took the CR of (26) to be as (23) and the causative morpheme -*sase* as the overt spell out of the CAUSE concept. Accordingly, they interpreted the verb as a lexical causative.

**Table:** 

(26)	Osarusan-ga hako-o **ak**-**e**-**sase**-ta-yo (Japanese).
	Monkey-NOM box-ACC open-TR-CAUS-PAST-PRT.
	“The monkey had [someone] open the box.”

In sum, the CR of lexical causative verbs includes at least two concepts, each introducing one argument. These two concepts are generally compressed, but sometimes children overtly articulate both and may interpret sentences as if the material expressed stems from a 1-to-1 mapping from the CR.

### 3.3. Antonymic concepts

The third domain is that of antonymic concepts, that is, concepts that can be expressed with several different grammatical categories and come in pairs, one member being positive and the other being its negative counterpart. This domain will be illustrated with data from prepositions (with vs. without), negative quantifiers and antonymic adjectives.

#### 3.3.1. Cum sine

By “cum sine” from Latin, we intend to refer to antonymic concepts that refer to the presence of something “cum” (positive member), expressed in English by “with” and the absence of something “sine” (negative member) expressed in English by “out.” We assume that while the positive member is a primitive, the negative one is complex and must be decomposed into “cum” plus a negative expression. Thus, the CR of “sine” is (27).

(27) CUM NEG

In languages like English, this CR is overtly realized into language with the morphemes “with-out.” In other languages, it is expressed as “CUM” plus sentential negation or a negative element; in yet other languages, it is a monomorphemic word that overtly and univocally expresses the absence, while the concept of presence is not articulated into language. This is the case in Italian or German, where the words, *senza*, *ohne*, are used respectively. Children, who may prefer to overtly realize all pieces of the CR are expected to produce “con senza,” “mit ohne,” thus making the mapping between the CR and language more transparent. And this is indeed the case as children have been found to produce sentences like (28) (see Sauerland et al., submitted).

**Table:** 

(28)	Schmeckt mir nicht	**mit** ohne Butter.	German
	Non mi piace	**con** senza Burro	Italian
	I don’t like it	**with** without butter.	

A survey we have done indicates that similar productions are found in child Dutch (met-zonder), Greek (me-ohi), Portuguese (com-sem), Shugnan (gati na), Basaa (ngi ni), Tagalog (may walang), Chabacano (tiene nuay), Indonesian (nggak pake). Although at this point, this evidence in only anecdotal and more investigation is needed (see Sauerland et al., submitted), we think that these data are highly suggestive and supportive of the idea of a CR as in (27).

#### 3.3.2. Antonymic adjectives

Antonymic adjectives are pairs like big/small, long/short, tall/little. Like for the prepositional antonym “cum sine,” one member of the pair is positive (e.g., big) and the other is negative (e.g., small). While the positive member of the pair is a primitive concept, i.e., it is not further decomposable, the negative member does not and is decomposed into two concepts comprising a negative concept and the corresponding positive one. Thus, the CR of “small” is.

(29) NEG BIG

As before, since children are biased to a 1-to-1 correspondence we predict that children may sometimes say “not big” rather than “small”; we also predict that acquisition of the positive member is easier than acquisition of the negative one. As for the first prediction, we do not know of any relevant evidence, while for the second, various investigations have been conducted which have led to different results. Some studies found that positive antonyms are mastered earlier than negative ones (Clark, 1972; Ehri, 1976; Barner and Snedeker, 2008) and others that they were mastered at the same time (Carey, 1978; Phillips and Pexman, 2015). No study has found that negative adjectives are mastered earlier than positive ones. In a recent study on Italian, Pagliarini et al. (2022) have probed the comprehension of antonymic adjectives, grande/piccolo (big/small), lungo/corto (long/short), alto/basso (tall/little) in three groups of Italian-speaking children aged 3, 4, 5-year-olds and one adult group. They used a card selection task with novel nouns. Children were asked to categorize into two sets 9 objects named after the novel noun according to whether they were considered big or small. The results showed that from age 4, but not 3, children and adults behaved the same with positive adjectives. However, for negative adjectives all children’s groups differed from adults, that is, even the 5-year-old’s comprehension of the negative antonym was not adult-like. This fact is compatible with the idea that the negative member of the antonymic pair is more complex than the positive one and in our framework that its underlying CR is as in (29).

#### 3.3.3. Negative concord sentences

Negative quantifiers, like *none*, stand in the antonym relation with existentials like *someone*. The antonym generalization, therefore, predicts that *none* and its counterparts across languages should be composed out of negation and an existential. Cross-linguistically the situation is more complicated because of the difference between double negation and negative concord structures. In Standard English, sentences like (30) featuring the presence of two negative elements yield a double negation reading, whereby the two negative elements cancel each other and the meaning of the sentence is equivalent to John bought something.

(30) John did not buy nothing.

By contrast, in languages like Italian, the two negative elements in the same structure, as in (31), give rise to a negative concord interpretation, whereby the two negative elements count as a single negation.

**Table:** 

(31)	Gianni non ha comprato niente.
	Gianni NEG has bought nothing.
	“Gianni bought nothing.”

Along with Bech (1955), Sauerland (2000b), Penka (2011), and Zeijlstra (2011) we assume that negative words, like *nobody*, *kein*, *nessuno* are decomposed into an Existential quantifier (positive quantifier) and negation, thus the CR containing a negative word is as in (32):

(32) NEG EXIST

A transparent expression of this CR is one in which NEG is expressed as sentential negation and EXIST is articulated as a quantifier, which in the context of negation would turn out to be a negative word, like *nessuno* (nobody) as in negative concord structures. In English, NEG is compressed (or not pronounced) and EXIST, being in the context of (covert) negation, is articulated with the negative word *nobody*. When EXIST is not in the context of the negation, it is articulated as *someone* (in both languages) [see Temmerman, 2012 for an alternative view, which builds on Bech, 1955 and which is likely incompatible with our proposal, as in our framework, this alternative would involve fusion of NEG and EXIST into a negative word plus addition of negation to obtain (31)].

Given the CR in (32), we expect children speaking languages in which two negative elements in the same structure lead to a double negation reading to be biased to produce negative concord sentences. Interestingly, English-speaking children produce negative concord sentences. Below are some examples from Adam and Sarah (Childes, MacWhinney, 2000) reported in Thornton et al. (2016):

**Table:** 

(33)	a. I didn’t do nothing (Adam 3;5) file 63.
	b. Because nobody didn’t broke (Adam 4;5) file 107.
	c. I don’t want to share none of my book (Sarah 4;6) file 49.
	d. I am not scared of nothing (Sarah 4;7) file 51.

In these examples, children map NEG to the negative morpheme (n’t) and express EXIST as a negative word (nothing, nobody) because it is in the context of negation, as we said earlier, i.e., they express (32) transparently. Furthermore, the negative concord interpretation seems to be the default for English-speaking children. Thornton et al. (2016) designed a Truth-Judgment value experiment in which they presented to 4;7-year-old children and adults sentences like (34), which could be amenable to a double negation or a negative concord interpretation.

(34) The girl who skipped didn’t buy nothing.

While adults generally attributed a double negation reading, children attributed a negative concord reading. Thus, unlike adults, English-speaking children seem to allow negative concord structures. In the same experiment, Thornton et al. (2016) established that children interpret as adults do sentences like (35), which are grammatical in the adult language.

(35) The girl bought nothing.

Furthermore, Nicolae and Yatsushiro (2020) investigated 4;2–6;5 (*M* = 5;2) year old Northern German–speaking children’s comprehension of negative concord sentences like (36):

**Table:** 

(36)	Der Hase hat kein Gemüse nicht gegessen.
	The rabbit has no vegetable not eaten.
	“The rabbit didn’t eat any vegetable.”

Standard German, and possibly the variety to which the children were exposed, is not a negative concord language, while dialects spoken in Southern Germany are, as is Austrian German. As in Thornton et al. (2016) German-speaking children assigned a negative concord reading to sentences such as (36), while adults a double negation reading. In addition, Nicolae and Yatsushiro (2020) report that German-speaking children from the age of 2 years produce negative concord sentences both in spontaneous speech (Childes, MacWhinney, 2000) and in experimental settings:

**Table:** 

(37)	Weiss nicht kein (Andreas 2;1).
	Know not kein.
	“don’t know any.”

Thus, children speaking languages where two negative elements in the same sentence lead to a double negation reading, English and German, produce negative concord sentences and assign a negative concord reading to sentences (34) and (36), as do children speaking a language where two elements in the same structure lead to a negative concord interpretation. In this respect, English and German-speaking children are unlike adults, and they behave in line with our hypothesis that they attempt to transparently produce and interpret sentences. One may object that English- and German-speaking children do not accept the double negation reading because it is difficult. First, this criticism can be leveled at the comprehension’s study, but not at the production of children’s negative concord sentences. Second, children, in Thornton et al. (2016) study, did not have any trouble in interpreting correctly sentences with two negative elements, as in (38), in which a negative concord reading is not possible because negation does not c-command the negative quantifier (see Zhou et al., 2014 for additional evidence from Mandarin):

(38) The mouse [who didn’t dressed up] cook nothing.

The fact that children can process two negative items in the same sentence weakens the criticism that children cannot process two negations, but this point is certainly one to keep in mind in future research.

So far, we have seen that English- and German-speaking children produce non-target negative concord sentences, interpret sentences with two negative elements as cases of negative concord. The third prediction of our approach is that when the CR is transparently expressed into language, abiding by the 1-to-1 mapping between the CR and the linguistic expressions, the acquisition of negative quantifiers should be easier. This prediction boils down to the fact that negative quantifiers should be easier to acquire in Italian than in English or German, as in the two last languages NEG is compressed. Katsos et al. (2016) examined the comprehension of negative quantifiers in 31 early languages and found earlier acquisition of negative quantifiers in negative concord languages than in non-negative concord ones, as our approach predicts.

### 3.4. Summary

In sum, we have illustrated three domains in which children lexicalize more concepts from the CR than adults do. These concepts that children produce are complex in that they can be decomposed into several primitive concepts, as several works in linguistics have conjectured. In overtly expressing them children attempt to express the CR as transparently as possible. At the same time, the 1-to-1 mapping that guides children non-target productions may guide children’s interpretation of sentences to the effect that these interpretations may differ from those assigned by adults (LD wh-questions, causatives, negative concord). Finally, structures that honor the 1-to-1 mapping facilitate language acquisition, as shown by the acquisition of the positive member of antonymic concepts or of negative quantifiers. In the next section, we are going to discuss alternative approaches to language acquisition and discuss why our is more promising.

## 4. Alternative accounts

Current research on child language based on the T-model of language has proposed three types of account for some specific features of child language that we discussed above, i.e., that children’s undercompression productions (1) have the same source of disfluencies widely studied in model of language production; (2) are due to general cognitive limitations on memory, attention (third factor) and (3) are the result of missetting a parameter and therefore are akin to a different adult language. Research within the usage-based paradigm would see undercompression errors as stemming (4) from the intrusion of frequent sequences when these are not appropriate (see McCauley et al., 2021). Before we address these views in more detail, note that the first three hypotheses are compatible with the undercompression view we espouse. In fact, in our view, undercompression errors are closely related to all three factors. However, none of the three hypotheses alone or together specifically predicts the undercompression errors we reported in section “3. Predictions for child language: undercompression errors” above.

### 4.1. Children’s commission errors and disfluencies

Let us start with the first account. We have been advocating a view in which children’s undercompression productions are revealing pieces of the CRs. However, we are aware that a potential competing view can be advocated that regards these productions as cases of disfluencies studied in the model of language production. Indeed, disfluencies have largely been investigated in adult psycholinguistic literature and have informed the architecture of models of language production (Lapointe and Dell, 1989; Levelt, 1989; Dell, 1995). Disfluencies include various types of errors, such as slips of the tongue, hesitations, self-corrections, omissions, repetitions, fillers (uhm). They display specific properties concerning the elements involved, and the processes that these undergo. For example, slips of the tongue may apply to different linguistic objects (such as phonological features, phonemes, morphemes, words, and phrases). These elements may be involved in various processes, but objects involved in a process must be at the same level (e.g., phonemes slip with phonemes) (Dell and Reich, 1981). These kinds of disfluencies have been taken as evidence for the different systems (monitor, planner) involved in the production of utterances. We acknowledge the possibility that not all children’s non-target production will be evidence for CRs, but which are and which are not is an empirical question that we are not able to disentangle at the moment. Even granting that some undercompression productions are disfluencies (see also section “4.2. Children’s commission errors and cognitive limitations”), the point is what the source of these disfluencies is. In the model of language production, one system is the conceptual system. We do not enter into the details of this system. But, to the extent that a message is conceived in the conceptual system and then expressed through language, we can say that our approach is not incompatible with models of language production. Our crucial hypothesis is that some undercompression sentences are evidence of richer structures built in the generator (in our system).

### 4.2. Children’s commission errors and cognitive limitations

As for the idea that children do not produce target-like structures because of cognitive limitations, again, we do not see any incompatibility with our approach. Our stance is that cognitive limitations may be responsible in some cases for children’s compression failures. However, cognitive limitations only give the framework for failing to produce an adult-compressed structure. What children produce is governed by the thought (generator) and linguistic (compressor) systems. To better understand how we approach cognitive limitations in our system, we discuss a recent proposal of one type of child’s error that, in our view, is revealing pieces of the CR, medial wh-questions, discussed in section “3.1.2. Long distance Wh-questions.”

Liter et al. (2022) have claimed that Long_Distance (LD)-wh-questions, as in (39) above and repeated below, are to be considered on a par with slips of the tongue, like “bad sack” pronounced as “bad back.”

(39) Which boy did you say who Mary called <which boy >?

Their explanation, based on Dell’s (1986) spreading activation model (see also section “4.1. Children’s commission errors and disfluencies”), is that the initial wh-element, pronounced in the first position, is highly activated and remains so, as it is needed to establish a dependency with the position it originates from [indicated in brackets in (39)]. Being highly activated, it may end up pronounced a second time in the intermediate position as a sort of perseveration error. In their view, children perseverate because they have poor inhibitory skills and cannot refrain from pronouncing the wh-element a second time in the intermediate position. Furthermore, they point out that slips of the tongue, in adults’ production, typically occur in legitimate positions and noticed that this property also holds of medial wh-questions. Children produce a second wh-element in the intermediate position, which is a legitimate position for wh-elements, as, e.g., in indirect questions. One potential problem for this account is that children may produce different combinations of wh-elements, as schematically shown in (40). Still, they never produce a copy of the sentence initial wh-phrase in the intermediate position (40d). Within Liter et al.’s framework, it could be claimed that only the + wh-feature is activated, and thus only a wh-word can be pronounced in the intermediate position. However, this would not explain the occurrence of (40b), in which the wh-phrase is in the intermediate position and *who* in initial position. Under our approach, the lack of (40d) stems from the fact that the features shared between the binder and the bindee (in a variable binding relation) must be those relevant for qualifying the relation (and the restriction N is not among those).

**Table:** 

(40)	a. Which boy…. Who…
	b. Who…… which boy….
	c. Who…. Who/where…
	d. *Which boy…… which boy…

A second problem is that in the adult literature on speech production, errors mostly involve content words (Garrett, 1975; Stemberger, 1985; Dell, 1990) and so (40d) would be expected, as a form of perseveration.

Under our view, the idea that inhibitory control limitations are at the heart of the phenomenon is not incompatible with our approach, as we merely say that children, because of some limitations to be investigated or because they do not know the rules of compression in their language, compress less than adults. What exactly they do when these limitations are operative is to find a solution that the thought and language system legitimate. In other words, the specific form of an undercompressed structure must be explained by appealing to these two systems and this is precisely what our approach has undertaken to do.

### 4.3. Parameter missetting

We turn now to the last alternative. Several of the child errors we discussed in section “3. Predictions for child language: undercompression errors” are fully grammatical in the adult grammar of another language. An alternative account in the T-model for such errors is that children have mis-set some parameters, and assume the grammar of a language where the “error” is fully grammatical (e.g., Yang, 2016). Such an explanation is particularly attractive where a variant of the language the child is learning actually allows the structure in question. For example, many varieties of English allow negative concord and therefore it is plausible that children may be exposed to a negative concord variety, even if the dominant variety in their environment doesn’t allow negative concord. Unlike the disfluency or cognitive limitations account, the parameter mis-setting account makes more concrete predictions concerning the errors to be found in children’s language. For any type of child error, it predicts there to be an adult language where that type of structure is grammatical. This prediction probably requires some further qualifications about how other cognitive limitations affect the prediction, for example, no adult language is at a one-word stage or lacks embedded clauses.

For a closer comparison of the predictions for child language, we can view the MFA as a specific subtype of parametric accounts. The MFA assumes that CRs are shared across languages and differences between languages derive from different compression, linearization and lexification rules. All possible constituent linearization statements and all concept articulation requirements could be viewed as binary parameters. Assuming this basis makes it possible to compare the undercompression and the parameter missetting accounts of child errors. This is especially so since linearization statements have been found to be acquired early, likely before children start to use multiword utterances (e.g., Christophe et al., 2003; Guasti, 2017 for review) and therefore language acquisition would be essentially restricted to learning compression parameters. On this view, the predictions of the MFA would mostly be inherited by a parameter-based model. One remaining difference is that on the parameter view, any undercompression ought to be possible, as a parametric option, in an adult language. But there are cases, in our view, where compression is explained by other properties of the language. As mentioned above, Sauerland et al. (submitted) discuss the case of the obligatory pronunciation of “with” in English “without” in comparison with German “mit ohne” which must be compressed to just “ohne” in the adult language (see section “3.3.1. Cum sine”). This variation does not seem to be one of parameter setting, but rather is rooted in the fact that “out” in English can express other meanings than the antonymic negation of “with.” Example (41) is a minimal pair where “out” and “without” both can occur, but express different meanings. Such examples don’t exist for German “mit ohne” and “ohne.”

(41) She had to stay somewhere outdoors/without doors.

For the MFA, this case, therefore, seems to toe the line between cases where compression is obligatory to occur in the adult language as in Dutch and German vs. a case where compression is blocked, i.e., English. Thus, children who produce “mit ohne” are not failing to compress because they produce something attested in some other language, but because they undercompress the two concepts, presence and absence. Thus, some undercompression errors are not necessarily possible options in some other grammar. Similarly, second language (L2) learners (adults and children) produce full nouns, rather than direct object clitic pronouns. Leonini and Belletti (2004) show that L2 adults of Italian (with different L1s) use lexical nouns in 40% of their production in contexts where the clitic pronouns would have been more appropriate (see also Belletti and Guasti, 2015 for review). Clearly, the use of lexical nouns rather than clitic pronouns does not fall under the scope of a parameter but can be easily accommodated by our approach as a failure of undercompression. Mantione (2016) found that 9-year-old children with Developmental Dyslexia and 4;4-year-olds typically developing children sometimes produce heavy prepositions rather than light prepositions. For example, they say (42a) rather than (42b) or (42c) rather than (42d). Notice that the choice of heavy prepositions cannot be attributed to phonological problems, because these participants also used light prepositions and the heavy and the light prepositions in (42) consists both of two syllables as the light ones include the articles.

**Table:** 

(42)	a. dentro la casa.
	inside the house.
	b. nella casa.
	in + the house.
	c. sopra la tavola.
	over the table.
	d. sulla tavola.
	over + the table.

Similar productions were also observed by Dal Farra et al. (2022b) in the spontaneous speech of Italian 2–3-year-olds children and can be seen as undercompression production.

In sum, there are cases of undercompression that do not necessarily fall under the scope of a parametric approach, but that can be seen as the results of undercompression. Therefore, the predictions of the MFA differ from the parameter mis-setting view.

### 4.4. Frequent sequences as the source of non-target productions

We turn now our attention to the last account of commission errors. In a recent paper, McCauley et al. (2021) have analyzed English-speaking children’s spontaneous production of failure to invert the subject and the auxiliary in non-subject wh-questions (data from Childes, MacWhinney, 2000), as in (43).

(43) What they are doing there?

We don’t think this type of non-target production reveals anything about CR, as all and only the ingredients necessary to form a question are present, and there is no undercompression. However, the proposal advanced by McCauley et al. (2021) can in principle, be extended to undercompression errors of the kind we have discussed. McCauley et al. (2021) operate under a usage-based approach, whereby humans store in memory words and multiword sequences with different degrees of abstraction and combine these sequences to form more complex ones. Multiword sequences compete and more frequent ones may be chosen during the production of a sentence, and may intrude, resulting in children’s universion errors in wh-questions. To support their proposal, McCauley et al. (2021) have extracted the wh-questions containing an auxiliary and featuring inversion or lack thereof and have computed the frequencies of n-grams forming the wh-questions (n-grams were sequences of words with *n* = 1, 2, 3). They have found that the frequencies of some n-grams predicted uninversion errors. For example, the more children hear “they are doing,” or “they are,” the more likely it is that they will produce “what they are doing?” and this may be expected. Frequency of multi-word sequences occurring later in the question, e.g., “doing there” predicted universion errors. Why this happens is less intuitive and remains to be clarified. Inversion was predicted by “what” and “are” but not by the sequence wh + Aux sequences, which would have been expected. This approach predicts which n-grams are responsible for universion errors, but it does not provide any explanation for why precisely those n-grams predict universion errors. Be as it may be, this approach could be applied to medial wh-questions. According to Dabrowska et al. (2009), non-target LD-wh-questions, (44a), are the result of the juxtaposition of two independent questions (44b) and (44c):

**Table:** 

(44)	a. Who do you think who Grover wants to hug?
	b. Who do you think?
	c. Who Grover wants to hug

But, we may note that (44b) cannot be an independent question and as such is ungrammatical, and (44c) is only possible as a subordinate clause. So, it seems that juxtaposition cannot do justice to the set of LD-wh-questions that children produce, and it remains to be seen which n-gram predicts (44a). We imagine that other non-target productions could be dealt in a similar way by appealing to n-gram frequencies predicting the “errors.” But once one knows which n-grams predict the “errors,” one may want to understand why precisely those n-grams and why those n-grams are more frequent in the first place, i.e., what is special about them that makes them more frequent. Without answering these questions, frequencies of n-grams are just a description but are not advancing our understanding of the structure and functioning of the mind. To appreciate this argument, consider studies by Changizi et al. (2006), Changizi and Shimojo (2008) on more than 100 writing systems. They found that the relative frequency of configurations of strokes is the same across writing systems. For example, “L” and “T” are more frequent than “X,” or “Y,” “F” is more frequent than “X” and so on. Thus, the frequency profile of these writing systems predicts that a newly invented writing system will not be different from those existing already, i.e., the configurations recruited in the newly invented system will not only be variants of those already present in other system, but will also have the same relative frequencies. But these facts wouldn’t tell us very much about humans’ minds, except that the inventor of the new system was very good at keeping track of relative frequencies. Changizi and Shimojo (2008) show that this regularity stems from constraints from our visual system. Those configurations have been culturally selected to exploit “what humans have evolved to be good at visually processing” (Changizi and Shimojo, 2008). In a nutshell, the selection depends on humans’ visual system. Similarly, some aspects of language are frequent because they depend on what humans are good at processing. To return to our point, if the frequency of n-grams can predict some undercompression errors, we would like to know why precisely those n-grams, what is special about them. On the MFA, children’s undercompression productions (at least some) stem from a richer conceptual structure and ensue from a failure of compression.

## 5. Conclusion

This article explores the predictions of the MFA for language acquisition. Our starting point is that language compress CRs and that children, while figuring out the compression function operative in their language, may undercompress and attempt to linguistically encode CRs through a 1-to-1 mapping, as stated in the Child Language Undercompression hypothesis (CLUH). These attempts will result in undercompression “errors.” We have discussed a number of undercompression errors found in the literature ensuing from the domains of variable binding dependencies, multi-argument verbal concepts and antonymic concepts. Children sometimes produce concepts staying for the binder and bindee, relative clauses with resumptive pronouns or DPs and long distance questions. Children may also decompose a given verb and produce one predicate per argument, and finally, they may produce negative concord sentences, even if the language does not feature it. In addition, children acquire more smoothly structures that map CRs in a transparent way; for example, they acquire the positive counterpart of antonymic adjectives before the negative one, or negative quantifiers earlier in languages in which negation is directly mapped to a linguistic expression (as in negative concord languages). Thus, there is some encouraging evidence in the literature for our view that undercompression errors may lead us to uncover the CR underlying our linguistic expressions. This evidence is far from being conclusive but sets our research agenda for a more systematic investigation in different linguistic areas.

## Data availability statement

The original contributions presented in this study are included in the article/supplementary material, further inquiries can be directed to the corresponding author.

## Author contributions

MTG drafted the manuscript along with US and AA commented on it. All authors contributed to the article and approved the submitted version.

## Footnotes

1 We use the term undercompression also to cover alternative ways of compressing than what is found in the adult language in the same circumstances. For example, Belletti and Manetti (2018) pointed out that Italian-speaking children use “si-causative passive” (where “si” is a reflexive clitic), as in (i), while adults do not. The structure used by children is available in the target language but not with a passive meaning, rather it has a causative meaning as in (ii) (see also Guasti et al., 2012 for another case in wh-questions).

**Table:**

(i)	Il bambino	si	fa	lavare dalla	mamma.
	The child	SI	makes	wash by-the	mommy.
	“The child	has mommy	wash him.”
(ii)	The child	made mommy	wash him. These alternative ways of compressing will not be dealt with in this article.

2 Overcompression is possible and an example is headlinese. This aspect will not be considered here.

3 A reviewer asks what are the predictions of the MFA for the acquisition of agglutinative, inflectional and isolating languages. Agglutinative languages may be easier to learn as they are more transparent, being characterized by separative morphology (see Wagner, 2021 for a review of the literature). However, when the mapping between concepts and morphemes is not 1-to-1 the child may have problems, but this would be the same in the three types of languages in so far as all three types of languages will compress CRs somehow. Rather, the problem would be one of morpheme segmentation in agglutinative and inflectional languages, i.e., to find the root and the various suffixes, prefixes. Proposals exist in the literature that take care of this problem (see Guasti, 2017 for review). This problem would be different in isolating languages, where concepts articulated otherwise through affixes could be articulated in a different way, if they will be articulated.
